# Which factors predict fertility intentions of married men and women? Results from the 2012 Niger Demographic and Health Survey

**DOI:** 10.1371/journal.pone.0252281

**Published:** 2021-06-09

**Authors:** Bright Opoku Ahinkorah, Abdul-Aziz Seidu, Eugene Budu, Ebenezer Agbaglo, Collins Adu, Kwamena Sekyi Dickson, Edward Kwabena Ameyaw, John Elvis Hagan, Thomas Schack

**Affiliations:** 1 School of Public Health, Faculty of Health, University of Technology Sydney, Sydney, NSW, Australia; 2 Department of Population and Health, College of Humanities and Legal Studies, University of Cape Coast, Cape Coast, Ghana; 3 College of Public Health, Medical and Veterinary Sciences, James Cook University, Townsville, Queensland, Australia; 4 Department of English, University of Cape Coast, Cape Coast, Ghana; 5 Department of Health Promotion, Education and Disability Studies, Kwame Nkrumah University of Science and Technology, Kumasi, Ghana; 6 Department of Health, Physical Education, and Recreation, University of Cape Coast, Cape Coast, Ghana; 7 Neurocognition and Action-Biomechanics-Research Group, Faculty of Psychology and Sport Sciences, Bielefeld University, Bielefeld, Germany; University of Salamanca, SPAIN

## Abstract

**Introduction:**

Niger is the country with the highest total fertility rate in the world. In the present study, we investigated factors associated with the desire for more children among married men and women in Niger.

**Materials and methods:**

We utilised data from the 2012 Niger Demographic and Health Survey. The outcome variable for the study was fertility intentions. The data were analysed with Stata version 14.0. Both descriptive (frequencies and percentages) and inferential (binary logistic regression) analyses were carried out.

**Results:**

Desire for more children was 97.2% and 87.2% among men and women respectively. Women aged 45–49 were less likely to desire more children, compared to those aged 25–39 [aOR = 0.13, CI = 0.11–0.16]. The odds of desire for more children were high in rural areas, compared to urban areas [aOR = 1.61, CI = 1.20–2.17]. Childbearing women with seven or more births were less likely to desire more children, compared to those with 1–3 births [aOR = 0.09, CI = 0.06–0.14]. Men aged 50–59 were less likely to desire more children, compared to those aged 25–39 [aOR = 0.13, CI = 0.05–0.35]. Men with secondary/higher level of education were less likely to desire more children, compared to those with no formal education [aOR = 0.24, CI = 0.11–0.52]. Childbearing men with seven or more births were less likely to desire more children, compared to those with 1–3 births [aOR = 0.06, CI = 0.01–0.30].

**Conclusion:**

This study shows high fertility desire among men and women in Niger. However, the prevalence of fertility desire among men is higher than that of women. A number of socio-economic and demographic factors were found to be associated with desire for more children among men and women in Niger. This calls for a collective effort to educate women and men in Niger on the negative consequences of rapid population growth and large family sizes.

## Introduction

Population dynamics is very important to the social, economic, and environmental development of any nation, including the attainment of the Sustainable Development Goals [1]. While rapid population growth may have some merits, such as boosting the availability of human resources, its negative effects seem to outweigh the positive ones [1]. For instance, high population leads to decline in per capita income if the economy is unable to catch up with the population growth rate [1]. Besides, high population growth puts pressure on essential services such as formal educational institutions and hospitals, and leads to high unemployment rates. More importantly, rapid population results from large family sizes, which in turn increases the risks of maternal and child mortality [2, 3]. Low fertility rates have been linked to enhanced empowerment status of women and help women to pursue higher education [4].

In view of the negative consequences of rapid population growth, low- and middle-income countries all over the world are adopting policies to check population growth. For instance, since 2002, the Nigerien government has been providing free contraceptives to its citizens [5]. As at 2015, more than 90% of least developed countries with rapid population growth had instituted policies to check the growth rate [1]. Fertility rates of most low- and middle-income countries are declining at a slower pace [6–8]. Some sub-Saharan African countries like Ethiopia, Rwanda and Malawi have experienced rapid fertility decline [9]. However, sub-Saharan Africa (SSA) has generally been the region with the highest fertility worldwide [10], with Niger being the nation with the highest fertility rate [11, 12]. Over the years, the total fertility rate of Niger has been high and stable. It rose from 7 in 1992 to 7.2 in 1998 and reduced to 7 in 2006. From 2006 to 2012, the total fertility rate of Niger again rose to 7.6 [13].

Research has revealed some association between fertility and other variables such as reproductive behaviour [14], contraceptive use [15, 16], unmet need for family planning [16], childhood mortality, intra-house bargaining power [17], and educational background [18]. Some studies have also focused generally on the predictors of fertility desire [19–22]. In the context of SSA, such studies have attributed increasing fertility levels to the desire for more children [21, 22]. What this suggests is that attempts at reducing fertility rates must focus on the desire for more children and its associated factors. Similarly, a plethora of literature highlight some other potential predictors of high fertility, including absence of strong family planning programs, unmet need for family planning and socio-economic characteristics [23, 24]. Studies have revealed husband’s desire for more children [25], child’s sex preference, professional status, wealth index, and residential places of women [26] as being associated with the quest for more children. Previous studies have focused on women empowerment and fertility preference in Niger and other sub-Saharan African countries [27], contraception demand and fertility [28] as well as fertility variability over time [29]. However, a study on predictors of fertility intentions using a recent national survey is yet to be conducted in Niger, despite the high total fertility rate in the country. In the present study, we investigated factors associated with the desire for more children among men and women in Niger. Identification of such factors will be helpful for the design and formulation of population programs and policies.

## Materials and methods

### Study design

The study utilised data from the 2012 Niger Demographic and Health Survey (NDHS). Specifically, we used data from the men and women’s file. The survey focuses on essential maternal and child health markers, including fertility intentions [30]. The survey employs a two-stage stratified sampling technique, which makes the survey data nationally representative [31]. A total of 2,186 childbearing men and 5,969 childbearing women aged 25–59 and 25–49 respectively who had complete information on fertility intentions constituted the sample size for the study. Thus, adolescents and young women aged 15–24 were excluded. The decision to exclude adolescents and young women was based on empirical evidence that in terms of fertility preference, young women (15–24 years) have been considered as the cohort that is just beginning childbearing and will have high desire for more children [32–35]. A detailed description of the NDHS has been presented in the final report which is also available online at https://dhsprogram.com/what-we-do/survey/survey-display-407.cfm. We relied on the “Strengthening the Reporting of Observational Studies in Epidemiology” (STROBE) statement in writing the manuscript. The dataset is freely available via measuredhs website at https://dhsprogram.com/data/dataset/Niger_Standard-DHS_2012.cfm?flag=0

### Variables

#### Outcome variable

The outcome variable for the study was fertility intentions. This was derived from the question “Would you like to have a (another) child, or would you prefer not to have any more children?” It had four responses: “want a (another) child,” “want no more,” “cannot get pregnant,” and “undecided” [36]. Our outcome variable was computed from two of these responses, namely “want a (another) child,” coded as 1, and “want no more,” coded as 0. Women and men who provided any other response (“cannot get pregnant” and “undecided”) were excluded. First, we excluded women who mentioned that they cannot be pregnant since their fertility desire have no relevance for subsequent fertility. We further excluded men and women who were undecided of their fertility preferences because of our interest in looking at respondents who provided definite responses (want another child or want no more) to the question.

#### Independent variables

Fourteen variables for women and thirteen variables for men were considered as explanatory variables in this study. For women, the explanatory variables were age, level of education, partner’s level of education, place of residence, wealth index, occupation, parity, frequency of reading newspaper/magazine, frequency of listening to radio, frequency of watching television, region, type of marriage, decision making capacity, and sex of household head. All these variables were considered as explanatory variables for the desire for more children among men, except partner’s level of education. These variables were considered because of their statistically significant relationships with fertility intentions in previous studies [20, 27, 37] and their availability in the dataset.

### Statistical analyses

We analysed the data with Stata version 14.0 (Stata Corporation, College Station, TX, USA). The analyses were done in two steps. In the first step, descriptive statistics (frequency and percentages) were used to describe the characteristics of the respondents and chi-square test of independence was used to examine the association between the independent variables and fertility intentions. All the variables that showed statistical significance (p<0.05) at the chi-square test were moved to the next stage which was the multivariable logistic regression analysis. We checked for multicollinearity using auxiliary R^2^ and no evidence of multicollinearity was detected. Group significance from LR test of significance was reported for variables with more than two categories. The results were presented as adjusted odds ratios (aOR), with their corresponding 95% confidence intervals (CIs) signifying their level of precision. Statistical significance was declared at p<0.05. Sample weight was applied and the survey command (svy) was used to account for the complex sampling design of the survey.

### Ethical approval

Ethical approval was obtained from the Ethics Committee of ORC Macro Inc. as well as the Ministry of Health of Niger. The DHS follows the standards for ensuring the protection of respondents’ privacy. ICF International ensures that the survey complies with the U.S. Department of Health and Human Services regulations for the respect of human subjects. This was an analysis of secondary data and, therefore, no further approval was required for this study since the data is available in the public domain at https://dhsprogram.com/what-we-do/survey/survey-display-407.cfm.

## Results

### Descriptive and chi-square test results

Table 1 presents the results on fertility intentions of childbearing women across the explanatory variables considered in this study. The chi-square test showed that age, place of residence, occupation, parity, frequency of listening to radio, frequency of watching television, partner’s level of education, region, and type of marriage had statistically significant associations with the fertility intentions of women. The overall desire for more children among childbearing women in Niger was 87.2%. Across the socio-demographic characteristics of women, the prevalence of women who wanted more children was high among women aged 25–39 (94%), women who lived in rural areas (87.6%), non-working women (87.5%), and women with 1–3 children (97.3%). In terms of frequency of listening to radio and watching television, majority of the women who desired more children never listened to radio (87.9%) and never watched television (87.2%). For level of partner’s education, place of residence, and type of marriage, high prevalence of desire for more children were recorded among partners with primary education (90%), women who lived in the Diffa region (93.9%), and women in monogamous marriages (87.1%).

**Table 1:** Distribution of fertility intentions across socio-demographic characteristics of childbearing women in Niger (weighted).

Variables	Weighted N	Weighted %	Fertility intentions		p-value
			Want another	Want no more	
**Age**					<0.001
25–39	4773	80.0	94.0	6.0	
40–49	1196	20.0	54.4	45.6	
**Educational level**					0.256
No education	5210	87.3	86.0	14.0	
Primary	512	8.6	87.9	12.1	
Secondary/higher	247	4.1	84.3	15.7	
**Place of residence**					<0.001
Urban	879	14.7	81.2	18.8	
Rural	5090	85.3	87.6	12.4	
**Wealth index**					0.052
Poorest	1173	19.7	87.2	12.8	
Poorer	1154	19.3	87.0	13.0	
Middle	1250	21.0	86.6	13.4	
Richer	1250	20.9	86.9	13.1	
Richest	1142	19.1	83.8	16.2	
**Occupation**					<0.001
Not working	4023	67.4	87.5	12.5	
Working	1946	32.6	83.5	16.5	
**Parity**					<0.001
1–3 births	1184	19.8	97.3	2.7	
4–6 births	2571	43.1	92.6	7.4	
7 or more births	2214	37.1	70.7	29.3	
**Frequency of reading newspaper/magazine**					0.138
Not at all	5815	97.4	86.2	13.8	
Less than once a week	78	1.3	80.0	20.0	
At least once a week	76	1.3	86.7	13.3	
**Frequency of listening radio**					0.007
Not at all	2076	34.8	87.9	12.1	
Less than once a week	1733	29.0	85.6	14.4	
At least once a week	2160	36.2	84.7	15.3	
**Frequency of watching television**					<0.001
Not at all	4633	77.6	87.2	12.8	
Less than once a week	667	11.2	84.9	15.1	
At least once a week	668	11.2	82.0	18.0	
**Partner’s education**					0.008
No education	4953	83.0	85.5	14.5	
Primary	593	9.9	90.0	10.0	
Secondary+	423	7.1	86.6	13.4	
**Region**					<0.001
Agadez	84	1.4	76.8	23.2	
Diffa	166	2.8	93.9	6.1	
Dosso	792	13.3	80.9	19.1	
Tahoua	1292	21.7	88.9	11.1	
Maradi	1340	22.4	86.9	13.1	
Tillaberi	819	13.7	89.1	10.9	
Zinder	1118	18.7	84.9	15.1	
Niamey	358	6.0	81.6	18.4	
**Type of marriage**					0.007
Monogamous	3527	59.1	87.1	12.9	
Polygamous	2442	40.9	84.6	15.4	
**Decision making capacity**					0.653
Not alone	5255	88.0	86.2	13.8	
Respondent alone	714	12.0	85.5	14.5	
**Sex of household head**					0.401
Male	5373	90.0	86.0	14.0	
Female	596	10.0	87.3	12.7	
**National**	5969	100	87.2	12.8	

Source: Niger Demographic and Health Survey, 2012.

Table 2 presents the results on fertility intentions of childbearing men across the explanatory variables considered in this study. The chi-square test results showed that age, educational level, place of residence, wealth index, parity, frequency of reading newspaper/magazine, frequency of watching television, and region had statistically significant associations with the fertility intentions of men. The overall desire for more children among childbearing men in Niger was 97.2%. In terms of age, level of education, place of residence, wealth quintile, and parity, the desire for more children was high among men aged 25–39 (99.4%), men who had no formal education (97.9%), those who lived in rural areas (98.3%), men of the middle wealth index (98.8%), and men with 1–3 children (99.7%). With frequency of reading newspaper and watching television and place of residence, majority of the men who desired more children never read newspaper/magazine (97.6%), never watched television (98.2%), and lived in the Diffa region (99.2%).

**Table 2:** Distribution of fertility intentions across socio-demographic characteristics of childbearing men in Niger (weighted).

Variables	Weighted N	Weighted %	Fertility intentions		p-value
			Want another	Want no more	
**Age**					<0.001
25–39	1061	48.5	99.4	0.6	
40–49	695	31.8	97.0	3.0	
50–59	430	19.7	90.6	9.4	
**Educational level**					<0.001
No education	1611	73.7	97.9	2.1	
Primary	354	16.2	97.8	2.2	
Secondary/higher	221	10.1	90.3	9.7	
**Place of residence**					<0.001
Urban	379	17.3	93.3	6.7	
Rural	1807	82.7	98.2	1.8	
**Wealth index**					<0.001
Poorest	368	16.8	98.4	1.6	
Poorer	395	18.1	97.7	2.3	
Middle	481	22.0	98.8	1.2	
Richer	480	22.0	97.4	2.6	
Richest	463	21.2	94.2	5.8	
**Occupation**					0.674
Not working	19	0.9	95.5	4.5	
Working	2167	99.1	97.0	3.0	
**Parity**					<0.001
1–3 births	611	28.0	99.7	0.3	
4–6 births	588	26.9	97.0	3.0	
7 or more births	986	45.1	94.9	5.1	
**Frequency of reading newspaper/magazine**					<0.001
Not at all	1925	88.1	97.6	2.4	
Less than once a week	186	8.5	95.5	4.5	
At least once a week	75	3.4	86.4	13.6	
**Frequency of listening radio**					0.304
Not at all	357	16.3	97.1	2.9	
Less than once a week	790	36.2	97.7	2.3	
At least once a week	1039	47.5	96.4	3.6	
**Frequency of watching television**					<0.001
Not at all	1392	63.6	98.2	1.8	
Less than once a week	480	22.0	97.8	2.2	
At least once a week	314	14.4	92.3	7.7	
**Region**					<0.001
Agadez	42	1.9	94.2	5.8	
Diffa	80	3.7	99.2	0.8	
Dosso	252	11.5	99.3	0.7	
Maradi	454	20.8	97.9	2.1	
Tahoua	425	19.4	97.8	2.2	
Tillaberi	315	14.4	97.1	2.9	
Zinder	448	20.5	96.3	3.8	
Niamey	169	7.7	91.2	8.8	
**Type of marriage**					0.542
Monogamous	1625	74.3	96.9	3.1	
Polygamous	561	25.7	97.4	2.6	
**Decision making capacity**					0.657
Not alone	535	24.5	97.2	2.8	
Alone	1651	75.5	96.9	3.1	
**Sex of household head**					0.489
Male	2166	99.1	97.0	3.0	
Female	20	0.9	94.1	5.9	
**National**	2186	100	97.2	2.8	

Source: Niger Demographic and Health Survey, 2012.

### Results of the multivariable logistic regression analysis

Table 3 presents the results on the predictors of desire for more children among childbearing women in Niger. The likelihood of desire for more children decreased with age, with women aged 45–49 less likely to desire more children, compared to those aged 25–39 [aOR = 0.13, CI = 0.11–0.16]. The odds of desire for more children were high in rural areas, compared to urban areas [aOR = 1.61, CI = 1.20–2.17]. Childbearing women with seven or more births were less likely to desire more children, compared to those with 1–3 births [aOR = 0.09, CI = 0.06–0.14]. Women who watched television at least once a week were less likely to desire more children compared to those who never watched television [aOR = 0.62, CI = 0.45–0.86]. With region, compared to Maradi, the odds of desire for more children were lower in all regions, except Diffa, with the lowest odds among women in Agadez [aOR = 0.26, CI = 0.17–0.40].

**Table 3:** Binary logistic regression analysis on predictors of wanting more children among women in Niger.

Variables	AOR	95% Confidence Interval		Group significance
		Lower bound	Upper bound	
**Age**				<0.001
25–39	1			
40–49	0.13***	0.11	0.16	
**Place of residence**				
Urban	1			
Rural	1.61**	1.20	2.17	
**Occupation**				
Not working	1			
Working	0.96	0.80	1.15	
**Parity**				
1–3 births	1			<0.001
4–6 births	0.32***	0.22	0.47	
7 or more births	0.09***	0.06	0.14	
**Frequency of listening to radio**				0.007
Not at all	1			
Less than once a week	0.93	0.73	1.19	
At least once a week	0.90	0.70	1.14	
**Frequency of watching television**				<0.001
Not at all	1			
Less than once a week	0.80	0.60	1.07	
At least once a week	0.62***	0.45	0.86	
**Partner’s education**				0.009
No education	1			
Primary	1.25	0.92	1.71	
Secondary+	1.04	0.73	1.49	
**Region**				<0.001
Agadez	0.26***	0.17	0.40	
Diffa	1.01	0.62	1.64	
Dosso	0.34***	0.25	0.46	
Maradi	1			
Tahoua	0.63**	0.46	0.87	
Tillaberi	0.86	0.62	1.20	
Zinder	0.49***	0.35	0.68	
Niamey	0.49***	0.32	0.74	
**Type of marriage**				
Monogamous	1			
Polygamous	0.99	0.83	1.20	
**Pseudo R**^**2**^	0.267			

* p<0.05

** p<0.01

*** p<0.001.

AOR = Adjusted Odds Ratio; 1 = reference category.

Source: Niger Demographic and Health Survey, 2012.

Table 4 presents the results on the predictors of desire for more children among childbearing men in Niger. Men aged 50–59 were less likely to desire more children, compared to those aged 25–39 [aOR = 0.13, CI = 0.05–0.35]. Men with secondary/higher level of education were less likely to desire more children compared to those with no formal education [aOR = 0.24, CI = 0.11–0.52]. Childbearing men with seven or more births were less likely to desire more children, compared to those with 1–3 births [aOR = 0.06, CI = 0.01–0.30].

**Table 4:** Binary logistic regression analysis on predictors of wanting more children among men in Niger.

Variables	AOR	95% Confidence Interval		LR test significance
		Lower bound	Upper bound	
**Age**				<0.001
25–39	1			
40–49	0.46	0.17	1.28	
50–59	0.13***	0.05	0.35	
**Educational level**				
No education	1			<0.001
Primary	0.91	0.37	2.19	
Secondary/higher	0.24***	0.11	0.52	
**Place of residence**				
Urban	1			
Rural	1.43	0.53	3.85	
**Wealth index**				
Poorest	1			0.003
Poorer	0.65	0.21	2.01	
Middle	1.16	0.34	4.01	
Richer	0.59	0.21	1.67	
Richest	1.35	0.38	4.83	
**Parity**				
1–3 births	1			<0.001
4–6 births	0.09***	0.02	0.43	
7 or more births	0.06***	0.01	0.30	
**Frequency of reading newspaper/magazine**				<0.001
Not at all	1			
Less than once a week	1.05	0.43	2.53	
At least once a week	0.56	0.22	1.42	
**Frequency of watching television**				<0.001
Not at all	1			
Less than once a week	0.88	0.39	1.99	
At least once a week	0.52	0.24	1.14	
**Region**				<0.001
Agadez	0.47	0.16	1.42	
Diffa	1.54	0.28	8.55	
Dosso	2.46	0.51	11.94	
Maradi	1			
Tahoua	1.20	0.39	3.66	
Tillaberi	0.83	0.29	2.38	
Zinder	0.58	0.22	1.54	
Niamey	0.29	0.10	1.86	
**Pseudo R**^**2**^	0.267			

* p<0.05

** p<0.01

*** p<0.001.

AOR = Adjusted Odds Ratio; 1 = reference category.

Source: Niger Demographic and Health Survey, 2012.

## Discussion

Rapid population growth generally has negative implications such as high unemployment rate, increasing family size, and higher risks of maternal and child mortality [2, 3]. However, the ultimate reason for rapid population growth is generally a high fertility desire [21, 22]. Therefore, in order to control population growth, it is important to focus on factors associated with desire for more children. In the present study, we examined the predictors of desire for more children among childbearing men and women in Niger. In the subsequent paragraphs, we discuss our findings, focusing on age of men and women, parity, place of residence, region, level of education of men, and women’s exposure to media (television). However, we first discuss the prevalence of desire for more children recorded in this study.

In terms of the national prevalence of desire for more children, the present study recorded 97.2% and 87.2% among men and women respectively. The observed prevalence of desire for more children in this population is higher than what has been reported in most studies [20, 38]. They are much higher than the prevalence of 60% recorded in Ghana [17] and 63.1% recorded in Rakai, Uganda [20]. The high desire for more children could be a contributory factor to the high fertility rate in Niger [39]. In the past three decades, the lowest total fertility rate of Niger has been 7.0, which has serious implications for high population growth [12, 13]. In 2003, an attempt by the Nigerien government to control population growth through free distribution of contraceptives was thwarted by women’s non-use of the contraceptives, which was often explained by their desire for more children [40]. As Potts et al. [40] noted, this desire for more children among women in Niger is often driven by family dynamics representing male interests. Our findings are in line with the regularity found in high fertility settings where women express a desire to cease childbearing more often than men [41–43]. In a context of low female empowerment [27], it is, therefore, critical for population policies in Niger to focus not only on women but also on their husbands, as both influence actual fertility behaviour.

The study revealed that desire for more children is associated with the age of men and women in Niger. As clearly shown in this study, older men and women were less likely to express a desire for more children, compared to their younger counterparts. A possible reason for this finding could be that those men and women have reached their reproductive goals [44], or have their desires restrained after reaching menopause. This finding is in line with previous studies in Turkey [45] and Ghana [46]. In giving reasons for this finding, Akonor [46] and Seannot and Yeatman [47], for instance, argued that younger women have more reproductive years to desire more children. Despite this, 90% of older men aged 40–49 still want more children compared to 54% of women 40–49. The possible explanation for the high fertility preference among men aged 40–49 compared to women of the same age category is their longer reproductive life [48]. Hence, men aged 40–49 are still in their reproductive ages while majority of women aged 40–49 are likely to have reached menopause and, hence, will have reduced desire for more children. Our findings suggest a need to engage men as an important target group in fertility regulation.

We found that desire for more children declines with increasing parity among men and women in Niger. Specifically, childbearing men and women with three or more births were less likely to desire more children compared with those with parity one. The possible reason is again that an increasing proportion of couples has reached their desired number of children. Different factors have been found to be associated with desired fertility such as norms around family size, expectations about childbearing from partners, the need to maintain stability in relationship as well as family, and community expectations [20]. Further research is needed to investigate this finding, specifically with regard to the higher desire for children of men. A large empirical literature has examined linkages between desire for more children and parity among women. For example, Dyer et al. [49] indicated that some women are divorced because they cannot have children. This finding is similar to previous studies by Ahinkorah et al. [38], Hacettepe University Institute of Population Studies [45] and Ocalan et al. [50], which showed that the desire for more children declined greatly with the increase in the number of children of a childbearing woman. Other studies that made similar observations include Adebowale et al. [51], Babalola et al. [52], Eklund [53], and De Silva [54] in Nigeria, China, and Sri Lanka.

Women who lived in rural areas had a higher likelihood of desiring more children, compared to urban dwellers. This finding is in line with previous studies in Uganda [55] and Ethiopia [56] which showed that urban residence is associated with lower fertility desires. According to Mahmud et al. [57], women in urban areas are more empowered with lower fertility desire relative to women in the rural areas. It is argued that rural settlers often marry at a younger age, which leads to the desire for more children [58]. Ifelunini et al. [59] also argues that rural settlers perceive children as wealth and labour force for their subsistence farming activities, which tends to increase their fertility desires [59].

Region of residence was recorded as a significant predictor of desire for more children in Niger. Specifically, compared to Maradi, the odds of desire for more children were lower in all regions, except Diffa, with the lowest odds among women in Agadez. Certain socio-cultural and economic characteristics present in Agadez could explain this finding. For instance, except in Niamey, Agadez is the region with the lowest prevalence of child marriage in Niger [60]. The low prevalence of child marriage means low fertility desire, as child marriage has been considered as a major factor in the desire for more children among women [61]. In terms of socio-economic factors, the Agadez region in Niger is the region with the second highest adult literacy rate and gross enrolment ratio, behind the Niamey region [62]. We recommend future studies to look at the reasons why the Niamey region has the lowest prevalence of child marriage and the highest literacy rates and yet women in the region are more likely to desire more children, compared to those in Agadez.

Exposure to mass media was associated with desire for more children among childbearing women in Niger. Specifically, women who watched television at least once a week were less likely to desire more children compared to those who never watched television. This finding corroborates the findings of previous studies on the relationship between mass media and fertility by building a smaller family ideal [38, 63, 64]. The possible reason for the finding is that messages on social media such as television have the possibility of influencing the fertility behaviour of women through the use of family planning methods and limit the number of children they wish to have. This can be seen through adverts on the beauty of small family size shown on television [65]. This finding indicates the importance of media exposure in fertility and calls for the possibility of educating on the advantages of small family size in Niger through the media.

We found that men with secondary/higher level of education were less likely to desire more children, compared to those with no formal education. This confirms previous findings that higher education is associated with lower fertility desire [66, 67]. A possible reason is the lower fertility goals of more educated men, that better appreciate the advantages of a small family and may perceive an additional child as a hindrance. This, however, is in contraposition to findings in other contexts of a positive association between male education and fertility desires [68–70]. These authors argue that more educated men have higher average earnings, making it possible to afford a larger family. Contrary to the findings of previous studies [38, 71, 72], the current study found no statistically significant association between the level of education of women and desire for more children. However, we found the highest prevalence of desire for more children among women with secondary/higher education in Niger. Although studies that have found lower odds of fertility desire among women with higher levels of education, compared to those with lower levels of education, have cited a number of mechanisms such as increased autonomy, exposure to new childbearing and gender norms, and increased employment opportunities as contributing to the low fertility desires [73–75], our finding of no significant association between female education and desire for more children illustrates the possible reasons for the extremely high fertility levels in Niger and calls for more research into the reasons for such a high fertility desire among educated women. Low statistical power could also account for the non-significant associations between level of education of women and fertility desire in the current study.

### Strengths and limitations of the study

The main strength of this study is the use of a nationally representative dataset that employed multi-stage sampling technique to select the respondents, which makes it feasible to generalise the findings to all childbearing men and women in Niger. The relatively large sample size also aided in fitting a robust logistic regression to model the factors associated with desire for more children while controlling for confounders. Despite these strengths, it is impossible to establish temporality of sequence and the possibility of social desirability biases cannot be overruled. Our analysis was also restricted to only variables available in the dataset. Other factors such as socio-cultural norms and values could not be accessed. The dataset is relatively old (10 years old) and, hence, may not provide the current situation of fertility intentions in Niger. However, that is the most recent DHS with available data in Niger. Another limitation is that fertility desires change along the life course [76, 77].

## Conclusion

This study has found high fertility desires among men and women in Niger. However, the prevalence of fertility desire among men is higher than that of women. A number of socio-economic and demographic factors were found to be associated with desire for more children among men and women in Niger. This calls for a collective effort to educate women and men in Niger on the negative consequences of rapid population growth and large family sizes. The population policies and programmes of countries such as Ethiopia, Malawi, and Rwanda, whose fertility rates have declined, can be adopted by government and non-governmental organisations in Niger as a guide in enhancing fertility decline in the country.

## Abbreviations

AOR: Adjusted Odds Ratio
CI: Confidence Interval
DHS: Demographic and Health Surveys
MMR: Maternal Mortality Ratio
WHO: World Health Organization
SDG: Sustainable Development Goal
SSA: sub-Saharan Africa
VIF: Variance Inflation Factor
